# P02.16. Fifteen minutes of yoga postures or guided meditation in the office can elicit psychological and physiological relaxation

**DOI:** 10.1186/1472-6882-12-S1-P72

**Published:** 2012-06-12

**Authors:** G Melville, D Chang, B Colagiuri, P Marshall, B Cheema

**Affiliations:** 1 University of Western Sydney, Sydney, NSW, Australia; 2 School of Psychology, University of New South Wales, Sydney, Australia

## Purpose

Psychological stress in the workplace is an independent risk factor for cardiometabolic diseases and related mortality. This exploratory study compared the effect of acute (15 min) yoga postures and guided meditation practice, performed while seated in the office workspace, on psychological and physiological markers of stress.

## Methods

A within-subjects crossover design was utilized. Each participant completed three conditions, including yoga postures, meditation, and control (usual work), separated by >24hrs. Perceived stress and blood pressure were evaluated before, immediately after, and at 3x 5-min intervals post intervention. Heart rate, respiratory rate and parameters of heart rate variability (HRV) were collected continuously, before, during and post intervention. Twenty adults (39.6±9.5yr) completed the study.

## Results

The yoga and meditation interventions significantly reduced perceived stress versus control. This effect was maintained throughout the 15-min post-intervention period. Yoga postures increased heart rate while meditation reduced heart rate versus control (both p<0.05). Respiratory rate was reduced during both yoga and meditation versus control (p<0.05). Time and frequency domains of HRV (i.e. SDNN and log-total power) were significantly improved during yoga versus control. Additional HRV outcomes (LF and LF:HF) indicated increased parasympathetic modulation during yoga versus control. Meditation improved HRV outcomes versus control only during the initial 5-minutes of the 15-min intervention period. All physiological parameters generally regressed to baseline during the post intervention period. Blood pressure indicated normotension during the baseline recording in all conditions and did not improve in yoga versus control. Meditation induced a reduction in both systolic and diastolic blood pressure at 5-min post intervention versus control (p<0.05).

## Conclusion

Yoga postures or meditation performed in the office environment can acutely improve several psychological and physiological parameters associated with the stress response. Use of such practical interventions to mitigate stress in the workplace may reduce the risk of cardiometabolic diseases and enhance job satisfaction and productivity.

